# Assessing the Health Risk and Trophic Transfer of Lead and Cadmium in Dairy Farming Systems in the Mantaro Catchment, Central Andes of Peru

**DOI:** 10.3390/toxics12050308

**Published:** 2024-04-24

**Authors:** Doris Chirinos-Peinado, Jorge Castro-Bedriñana, Eustace P. G. Barnes, Elva Ríos-Ríos, Edgar García-Olarte, Gianfranco Castro-Chirinos

**Affiliations:** 1 Nutritional Food Safety Research Center, Universidad Nacional del Centro del Perú, Huancayo 12007, Peru; dchirinos@uncp.edu.pe (D.C.-P.); egarcia@uncp.edu.pe (E.G.-O.); 2 Cavendish Laboratory, University of Cambridge, JJ Thomson Avenue, Cambridge CB3 0HE, UK; eustacepgbarnes27@gmail.com; 3 Science Faculty, Universidad Nacional Agraria La Molina, Lima 15024, Peru; erios@unalm.edu.pe; 4 Faculty of Psychology, Universidad Peruana de Ciencias Aplicadas, Lima 15054, Peru; u201511072@upc.edu.pe

**Keywords:** heavy metals, raw milk, potential risk, contaminated food chain, food toxicology, target hazard quotient

## Abstract

This study investigated lead (Pb) and cadmium (Cd) transfer in three dairy farming areas in the Mantaro river headwaters in the central Peruvian Andes and at varying distances from the mining complex at La Oroya. At each of these sites, the transfer of trace metals from the soil to raw milk was estimated, and a hazard assessment for lead and cadmium was carried out in scenarios of minimum, average, and maximum milk consumption in a Peruvian population aged 2–85. Pb and Cd were quantified by flame atomic absorption spectrometry. Significantly, the concentrations of lead and cadmium were found to exceed the maximum limits recommended by the World Health Organization, with a positive geospatial trend correlated with the distance from mining activity. Both Pb and Cd were found to be transferred through the soil–pasture–milk pathway, with the primary source of Cd being phosphate-based fertilizers used in pasture improvement. Pb was found to be the most significant contributor to the Hazard Index (HI) with those under 19 years of age and over 60 recording an HI of >1, with infants being the most vulnerable group due to their greater milk consumption in relation to their body weight. A marginal increase in contamination was observed in the dry season, indicating the need for studies to be expanded over several annual cycles.

## 1. Introduction

The milk of cattle is recognized as being a good source of energy, water, carbohydrates, fats, proteins, amino acids, sugars, vitamins, minerals, and enzymes [1]. It is generally considered to improve child growth and development [2] and contains bioactive elements related to early metabolic development related to the epigenetic signaling processes [3]. Its high intake is associated with protective effects against a wide variety of health conditions [4], and, as such, the safety of such foodstuffs should be accorded the highest priority [5]. The production of milk of the best chemical, microbiological, and toxicological quality is essential for both the general health of the population and child development. Elevated levels of Pb and Cd are particularly known to impact child development and increase the incidence of a wide variety of health conditions [6].

Scientific research has established the presence of heavy metals in milk in varying concentrations associated with intensive agriculture, industrialization, and urbanization [7]. Lead (Pb) and cadmium (Cd) are ecotoxic compounds that contaminate the soil and the products generated in this substrate [8,9,10]. The dispersion and concentration of trace metals and persistent residues are known to contaminate water and soils, readily transfer to grassland species in pastures, with high levels of biomagnification in grasses [11], and transfer through the food chain [12,13] to plants [10,14,15], animals, and their tissues and products [16], including human populations. Due to their environmental persistence, trace metals are considered toxic to both ecosystems and public health [14,17,18], as they adversely affect food quality [19], environmental health, and human health, damaging a series of tissues, organs, and systems, affecting intellectual development, and causing a series of cancers [20,21,22,23,24,25,26,27,28,29]. 

Since Pb and Cd are non-essential and have no biological function, they are harmful even in low concentrations [30] and can cause severe carcinogenic and non-carcinogenic health risks when consumed above the acceptable limits recommended by international regulatory bodies [31]. This is recognized as a global problem of great concern [32]. As such, monitoring heavy metal concentrations in milk and the associated risk assessment is vital for maintaining product quality and is considered an essential statutory public health responsibility [33].

In the central Peruvian Andes, La Oroya, one of the most polluted cities on the planet, has been the center of polymetallic metallurgical complexes for over 100 years, although there are few environmental impact studies and little attention paid to the risk of bioaccumulation in milk products from the surrounding areas [13,34,35,36], especially in local populations, with little epidemiological and scientific evidence on the state of contamination of the soil–pasture–milk system and risk due to milk consumption. Specifically, the middle basin of the Mantaro River has an agricultural vocation and is the major supplier of milk and dairy products to the great city of Lima, from which processed products go to the entire country. This investigation is essential for statutory bodies to develop and apply regulatory processes, essential for both environmental and human health, which are based upon scientific evidence.

Globally, regulatory bodies have established maximum limits for Pb and Cd in milk and dairy products, and there is an urgent need to evaluate the safety of milk consumed by children. This study hypothesizes that the levels of Pb and Cd in milk produced in the middle basin of the Mantaro River, located in the high central Andes of Peru, would be high because of elevated levels found in both soil and pastures from mining and processing activity close to the production areas. The study objectives were to determine the levels of Pb and Cd in soils, pasture crops, and milk, assess them against internationally recommended limits, evaluate the exposure and health risk derived from milk consumption in three intake scenarios (minimum, average, and maximum) in a Peruvian population aged 2–85 years, and generate toxicological data for the region to assist with the development and implementation of mitigation strategies and the prevention of pollution in the environment and human health.

## 2. Materials and Methods

### 2.1. Study Area

**Figure 1:**
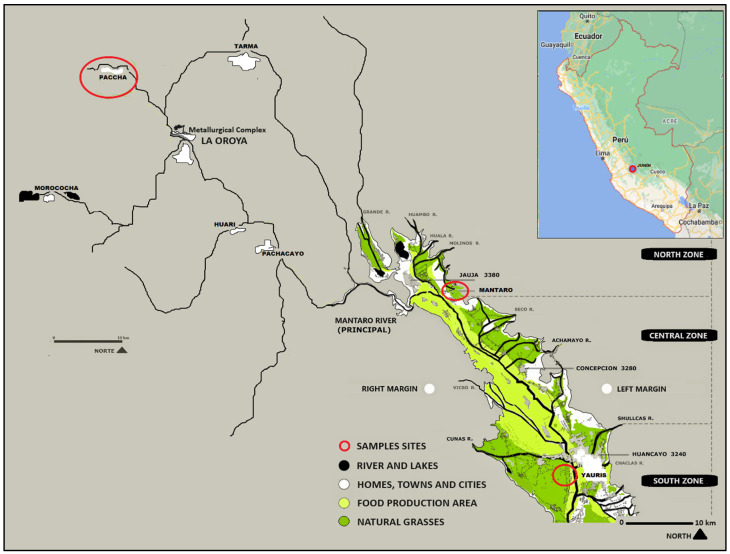
Relative positions of the Paccha, Mantaro, and Yauris sample sites and both regional urban centers and the Oroya mining complex in the Mantaro River headwaters, department of Junín, Peru. This region is in the high Andes, connected to Lima to the west, Huancayo to the south, Junín to the north, and Tarma to the east.

This study was carried out in March (wet season) and August (dry season) during 2023. The samples were collected from 3 zones in the Mantaro River middle basin: Paccha, Mantaro, and Huancayo (Junín-Perú), located 20, 92, and 124 km from the polymetallic metallurgical complex of La Oroya. (Figure 1). The community of Paccha (11°31′03″ S, 75°53′58″ W, 3742 m asl, and temperatures ranging from −3.1 to 18.2 °C) is located approximately 20 km from the metallurgical complex of La Oroya where copper, zinc, silver, lead, indium, bismuth, gold, selenium, tellurium, and antimony are processed. Mantaro (11°49′19″ S, 75°23′32″ W, 3353 m asl, with temperatures ranging from 1.2 to 18.0 °C) is an agricultural and livestock area located 83 km from La Oroya, and Huancayo was the third zone which was studied (12°4′36.3″ S, 75°13′30.2″ W, 3214 m asl, with temperatures ranging from 7 to 21 °C).

The latter is a densely populated city, located 123 km from La Oroya. The farmed area included in this study is adjacent to a mineral processing plant, which has operated for 60 years, and a disused municipal solid waste disposal site. The climate of the study areas is characterized as having two seasons, the wet season that generally lasts from October to March and the dry season from April to September. The Köppen and Geiger climate model classifies the regional climate as (ET) polar tundra, while the region is known as being in the Andean Puna grassland biome.

### 2.2. Sampling

At each of the three sites, samples of raw milk were taken from 20 lactating Brown Swiss cows between their third and fourth calving, totaling 60 cows. Samples were collected from each site during both the wet and dry seasons, giving 40 samples for each site and a total of 120 samples during the period of study, following the Peruvian Technical Standard [37], using 250 mL polyethylene bottles of first use and preserving the cold chain with dry ice. Twenty pasture 0.2 kg samples and 20 soil samples of 0.5 kg each were collected from each farm following standardized procedures [38,39,40]. Soil samples were taken at depths of 0–30 cm at 20 random points. Grass samples were collected from the same soil sampling sites. These samples were placed in polyethylene bags and stored at −4 °C until they were sent to the laboratory.

### 2.3. Sample Preparation and Metal Analysis

After a day of natural drying, the soil samples were crushed, sieved (2 mm mesh), and homogenized, removing gravel, stones, and other matter. They were then weighed and placed in airtight bags for shipment to the laboratory, where standard analytic procedures were followed, USEPA 3050B (SW-846), by digestion of 1 g of the dried sample treated with HNO_3_ (Sigma-Aldrich, St. Louis, MO, USA), H_2_O_2_, and HCl (Sigma-Aldrich, St. Louis, MO, USA). The grass samples were washed with tap water to remove soil particles, rinsed with deionized water [41], dried at 70 °C, and finely ground. The digestion and quantification of heavy metals in vegetative materials was the same as for soils samples and as described in earlier studies [42].

The milk samples were incinerated in a muffle oven (Protherm 442-ECO110/15) at 450 °C for 6 h, and, after cooling to 16 °C, they were treated with 2 mL of 2 N HNO_3_ (154.04 g/mol); after evaporation and cooling, they were incinerated at 450 °C/1 h. The ashes were recovered with 20 mL of 0.1 N HNO_3_ (7.70 g/mol), filtered through Watman No. 40 paper, and stored in polypropylene tubes under refrigeration. The procedure followed is detailed in Chirinos et al. [42]. The Pb and Cd quantification was carried out in a flame atomic absorption spectrometer (NAMBEI AA320N) following the AOAC 973.35 method, using wavelengths of 283.3 and 228.8 nm for Pb and Cd, respectively [43,44]. The analyses followed validated and standardized laboratory protocols accredited by the National Institute of Quality of Peru. Duplicate samples allowed for the determination of the precision method and the calculation of the mean and the coefficient of variation, which was less than 5%. Precision was measured using standard solutions of each element, determining the relative error, which in percentage represents the precision method and must exceed 95%. For these calculations, standard solutions of Pb and Cd of 155 and 150 mg/kg of milk were used, and at analysis, the corresponding concentrations were 148.14 and 152.50 mg/kg, values that, when transformed to a percentage, indicate that the method complies with the precision parameters. Calibration curves were prepared from a stock solution with Merck standards of 1000 mg/kg for each element. The limits of detection (LODs) of Pb and Cd in milk were 0.03 and 0.03 μg/L; the LODs for forage were 2.40 and 0.40 μg/kg, and the LODs for soil were 0.1 and 0.01 mg/kg, respectively.

To evaluate the concentration of Pb and Cd in the soil, the National Standard Environmental Quality Standard of Peru, of 70 and 1.4 mg/kg, respectively, was applied [45]. The Maximum Permissible Limit (MPL) of Pb used for forage was 10 mg/kg dry matter [46,47,48], and for Cd, it was 1 mg/kg [49]. The MPL for Pb in milk, established by the Codex Alimentarius Commission and the standard of FAO W [50] and the European Union [51], is 0.02 mg/kg. The MPL for Cd in milk established by the International Dairy Federation is 0.0026 mg/kg [52]. The concentrations of heavy metals in all study samples are expressed in mg/kg.

### 2.4. Transfer of Pb and Cd from Soil to Pasture and Milk

To evaluate the Pb and Cd transfer, the relationship between the metal concentration of the dry matter of the grass and the concentration of the same metal in the soil was estimated [53].

$$TF=\frac{CMpasture}{CMsoil}$$

where TF is the transfer factor, *CMpasture* is the metal concentration in the grass, and *CMsoil* is the metal concentration in the soil in mg/kg. 

The Bioaccumulation Factor (BF) is the ratio of milk metal concentration (*CMmilk*) and the soil concentration [54]:

$$BF=\frac{CMmilk}{CMsoil}$$

### 2.5. Risk Assessment

The weekly intake (WI) was used to assess heavy metal intake; the Target Hazard Quotient (THQ) was used to assess potential non-carcinogenic effects associated with long-term exposure to heavy metals in milk, and the Hazard Index (HI) evaluates the chronic risk of multiple heavy metals [55,56,57].

An exposure assessment was carried out for a Peruvian population aged 2 to 85 years using the average levels of Pb and Cd in milk from the studied areas and the corresponding milk consumption rates in three scenarios by age, minimum, average, and maximum consumption, estimated from various studies with Peruvian population data [18,58,59,60,61] and body weights from a single national study that included 62,600 people from 2 to 85 years old whose data are relevant, as there is no other study of this magnitude [62]. The daily milk intake estimated for this study as a minimum, average, and maximum level in children from 2 to 5 years old was 400, 500, and 600 g/day; for those from 6 to 11 years old, it was 480, 600, and 720 g/day, for those 12 to 19 years old, it was 500, 600, and 720 g/day, for those aged 20 to 59 years, it was 100, 150, and 240 g/day, and for those aged 60 to 85, it was 125, 185, and 290 g/day. With this information, a table of body weights and milk intake was prepared for ages 2 to 85 years, with continuous data to perform the calculations and create the corresponding risk graphs.

The daily intake (EDI) of Pb and Cd from milk consumption, in µg/kg body weight/day, was determined using the formula recommended by different authors [63,64,65]:

$$EDI=\frac{DFC\times MC}{WB}$$

which includes the following:

*DFC* is the daily milk consumption, in kg;
*MC* is the average metal concentration in the milk sample (mg/kg);
*WB* is body weight in kg.

The weekly intake (WI) was determined by multiplying the EDI by seven to assess against the internationally recognized tolerable weekly intake (TWI), which for Pb and Cd is 25 and 5.8 µg/kg BW [66,67,68,69].

The THQ and HI for Pb and Cd, for milk consumption, toxicologically indicate whether milk produced in the Mantaro River headwaters is within the levels established by the Codex Alimentarius [50] and avoids the risk of these metals for human health [66,67,70]. The potential non-carcinogenic chronic risk of heavy metals expressed as the TQH was calculated as follows [65,71]:

$$TQH=\frac{EF\times ED\times DFC\times MC}{RfD\times WB\times TA}$$

The Exposure Frequency (EF) and the exposure period equivalent to longevity (ED) for an adult are considered to be 365 days a year and the years studied (70 years). The following aspects were considered in the calculation:

*DFC* is the daily feed consumption (milk in kg).
*MC* is the average metal concentration in milk (µg).
RfD is the reference oral dose of each metal. For Pb and Cd, the values are 0.0035 and 0.001 mg/kg body weight/day, respectively [72,73,74].
*WB* is body weight in kg.
*TA* is the average lifespan in days, which is 25,550 days (70 × 365).

The HI assesses the potential long-term risk to human health when two or more heavy metals are involved. It is calculated as the sum of the THQ [75,76] and indicates the probable risk of contracting non-cancerous diseases. A value > 1 indicates a potential risk of health effects. If the HI is <1, no adverse health effects are expected due to exposure [77].

### 2.6. Data Analysis

Data were analyzed using SPSS 23 (IBM, Endicott, NY, USA). The results were expressed as mean ± standard deviation (SD). In the Kolmogorov–Smirnov test, the data did not follow a normal distribution, so the concentrations of Pb and Cd in the soil, pasture, and milk were compared using non-parametric Kruskal–Wallis tests. Differences were considered statistically significant when *p* < 0.05.

The Maximum Permissible Limits (MPLs) for Pb in soil, pastures, and milk are 70, 30, and 0.02 mg/kg, and for Cd, they are 1.4, 1.0, and 0.0026 mg/kg [51,78].

## 3. Results

### 3.1. Pb and Cd Concentrations in Soil, Pasture, and Milk

The average concentrations of Pb and Cd were calculated for soils and pastures used by dairy cattle, where Pb was the more prevalent of the two. The average concentrations of these heavy metals in the raw milk produced in the study areas were above the MPLs established internationally (Table 1).

### 3.2. Weekly Intake (WI) in a Peruvian Population Aged 2–85 Years Due to Consumption of Milk Produced in the Mantaro River Middle Basin

As an example, Table 2 shows the EDI and WI values for milk consumption in the minimum intake scenario as an easy-to-view summary.

**Figure 2:**
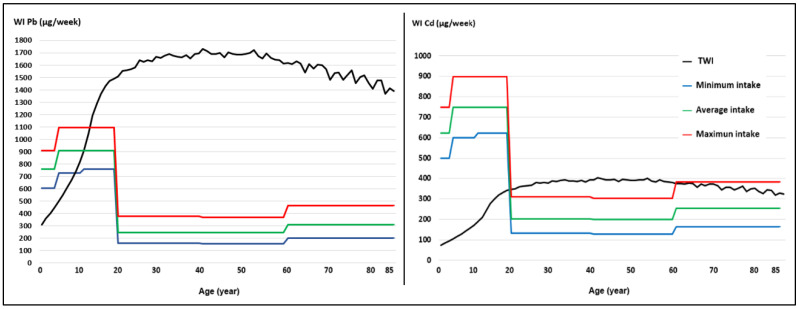
Weekly intake (WI) of Pb and Cd by minimum, average, and maximum consumption of milk produced in the Mantaro River headwaters, in the local population aged 2–85 years. The highest WIs were observed with the maximum milk intake level.

Figure 2 shows the weekly intake (WI) Pb and Cd curve for minimum, average, and maximum milk consumption produced in the Mantaro River headwaters in the local population aged 2–85 years. The WI was highest in the maximum milk intake level.

At the level of maximum milk intake (278 mL/day), it was observed that those over 60 years of age also exceed the TWI for Cd and would also be at potential risk of chronic Cd toxicity from milk produced under the study area conditions.

### 3.3. Target Hazard Quotient (THQ) and Hazard Index (HI) in a Peruvian Population Aged 2–85 Years Due to Consumption of Milk Produced in the Mantaro River Middle Basin

Table 3 summarizes the THQ and HI values in the minimum intake scenario. Continuous data for a population aged 2–85 years for minimum, medium, and high milk consumption were used.

**Figure 3:**
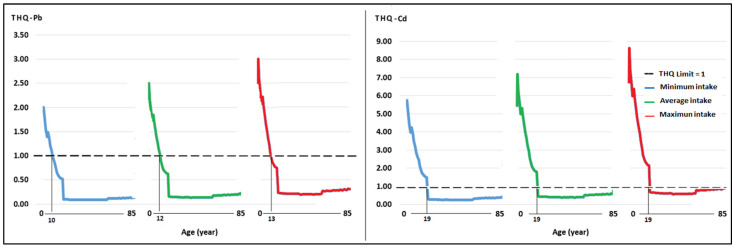
Average objective risk coefficient (THQ) for Pb and Cd for minimum, average, and maximum consumption of milk produced in the Mantaro River headwaters in the local population aged 2–85 years.

In the minimum, average, and maximum milk intake scenarios, the average THQ for Pb exceeded the value of 1 in people under 10, 12, and 13 years of age. The average THQ for Cd had values greater than 1 in people aged 2–19 (Figure 3).

**Figure 4:**
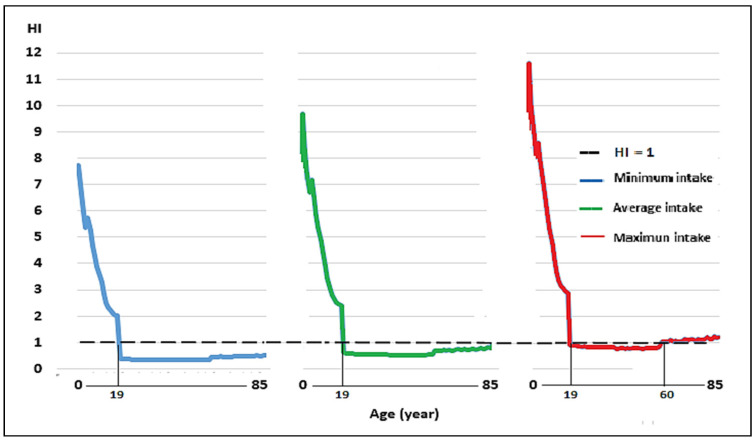
Hazard index (HI) for minimum, average, and maximum consumption of milk contaminated with Pb and Cd, produced in the Mantaro River headwaters, in the local population aged 2–85 years.

Regarding the HI, THQ Pb, and Cd sum, for the minimum and average milk consumption scenarios, the average value exceeded 1 in people under 2–19 years of age. In the scenario of maximum milk intake, an HI > 1 was observed in people over 60 years of age (Figure 4).

### 3.4. Objective Risk Coefficient (THQ) and Risk Index (HI) in the Peruvian Population Aged 2–85 Years Due to Consumption of Milk Produced in Three Central Highlands Zones in Dry and Rainy Seasons

**Figure 5:**
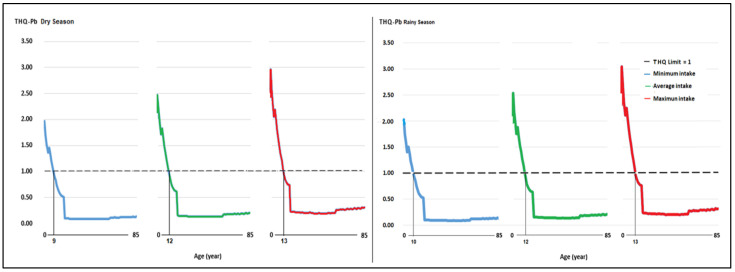
Objective risk coefficient (THQ) for Pb for minimum, average, and maximum consumption of milk produced in three areas of the central highlands in a Peruvian population aged 2–85 years in the dry and rainy seasons.

In the scenarios of minimum, average, and maximum milk intake, in the dry season, the THQ for Pb exceeded the value of 1 in people under 9, 12, and 13 years of age, while in the rainy season, the values exceeded 1 in people aged 10, 12, and 13 years (Figure 5), indicative of similar Pb contamination in milk at both times of the year.

**Figure 6:**
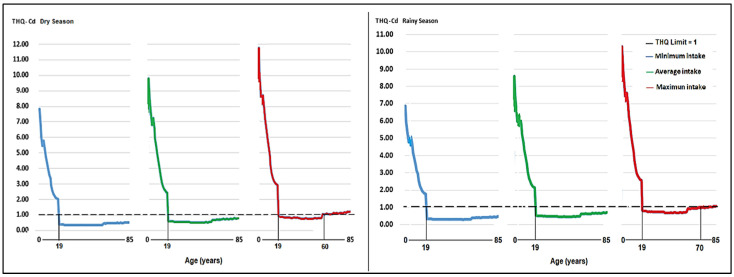
Objective risk coefficient (THQ) for Cd for minimum, average, and maximum consumption of milk produced in three areas of the central highlands in a local population aged 2–85 years in the dry and wet seasons.

In the Cd case, in the dry season, the THQ for Cd, in the three consumption scenarios, exceeded the value of 1 in people under 20 years of age. Regarding older adults, the THQ was greater than 1 in those over 60 years of age in the dry season and in those over 70 years of age in the wet season (Figure 6), which would indicate a similar level of Cd contamination throughout the year.

**Figure 7:**
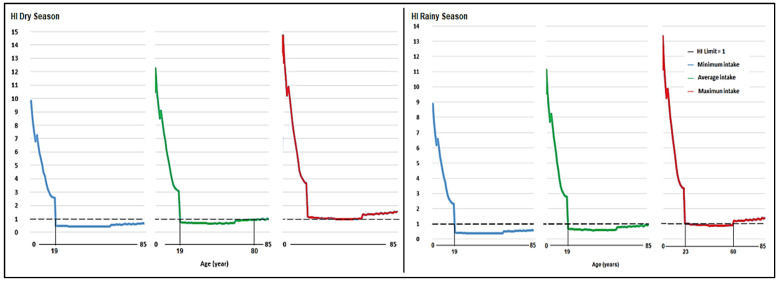
Risk Index (HI) of Pb and Cd for minimum, average, and maximum consumption of milk produced in three areas of the central highlands, in a local population aged 2–85 years, in the dry and wet seasons.

Regarding the Hazard Index (HI), which represents the danger of accumulated Pb and Cd in the milk produced in the Mantaro Valley headwaters in the dry and rainy seasons, in the scenario of minimum milk consumption, the value was greater than 1 in those under 19 years of age (Figure 7).

In the average intake scenario, the HI, in addition to those under 19 years of age, was also greater than 1 for people over 80 years of age, and in the maximum milk consumption scenario, the HI was greater than 1 in all ages; a result that would be indicative of a slightly higher level of contamination in the dry season, since in the wet season, in the scenario of maximum milk consumption, the HI was greater than 1 in people under 23 years of age and in those over 60 years old, which would show a slight increase in pollution in the dry season.

## 4. Discussion

### 4.1. Lead and Cadmium Levels in Soil, Pasture, and Fresh Milk Produced in the Mantaro River Headwaters

In this study, we evaluated the Pb and Cd concentrations in the soil, pastures, and raw milk produced at three sites in the Mantaro River headwaters (Table 1). The MPLs for Pb and Cd in soil are 70 and 1.4 mg/kg [45,51,78], and in this study, the average concentrations of these metals were 3.7 and 3.9 times higher than these limits. The Pb and Cd levels in soil ranged from 40.79 to 879.00 and from 0.002 to 18.21 mg/kg, indicating that the soils studied are highly contaminated.

The Pb content in the soil and pastures was similar to that reported in a livestock area near the La Oroya metallurgical complex, where 218 mg/kg was found in the soil and 20 mg/kg in the pastures [79]. The world literature indicates that the content of heavy metals responds to the environmental conditions of each region and country [7,10,40,80,81,82,83,84], and on a global scale, Peru is located at the top [42]. The main source of Pb contamination would be from irrigation water from the Mantaro River and fine particle emissions from the mining–metallurgy industry, descending onto pastures from atmospheric loading derived from mining activity and ore processing [85]. Regarding Cd, it was observed that the principal source of pollution comes from phosphorus-based fertilizers, commonly used in agricultural systems in the Peruvian central highlands [40]. In European countries, where there is greater control of industrial emissions, lower levels of heavy metal contamination have been reported [10,40,81,86,87].

The MPLs for Pb and Cd in grasses are 10 and 1 mg/kg [51,78], and in this study, the average concentrations of these metals were 1.4 and 1.2 times higher than these limits. The Pb and Cd concentrations in pasture range from 1.51 to 34.00 and ND to 6.53 mg/kg, indicative of high variability in the study sites.

The MPLs for Pb and Cd in milk are 0.02 and 0.0026 mg/kg [51,78,88,89], and in this study, the average concentrations of these metals were 10.9 and 68.5 times higher than these limits. A significant and very alarming result, considering the potential health risks associated with these trace metals for human health [83]. The ranges of Pb and Cd levels in milk were 0.001 to 0.60 and 0.001 to 0.69 mg/kg, respectively. The concentration of these toxic elements in order of importance is Pb > Cd, representing 55 and 45% of the total, respectively. These high levels of heavy metals, especially Cd, would be related to their presence in the soil and pastures due to the use of phosphorus agrochemicals [90], which contain up to 200 mg Cd/kg P_2_O_5_, with a moderate correlation (r = 0.66) between the application of phosphate fertilizers and Cd accumulation [40]. A second source of Cd contamination is from irrigation waters. Higher values than those determined in our study have been reported in China [91].

In a global review on the content of heavy metals in milk [7], Pb contents in raw cow’s milk below the detection level up to 60 mg/L were reported in a mining area in India. The accumulation of heavy and toxic metals in milk and derivatives depends on the proximity of the farms to the emission areas [27,81,83,91,92,93,94,95,96]. A higher concentration of Pb was observed in the milk from the farm closest to the mining activity, being 0.58 mg/kg in Paccha, which is located 20 km from the largest mining complex in Peru, 0.06 mg/kg in the farm located close to a mini mineral concentrator, and 0.015 in the El Mantaro farm located 92 km from the metallurgical complex. Regarding Cd in milk, the Paccha and Yauris areas showed lower values than Mantaro, a site where high phosphorus fertilization is used. It is worth mentioning that the main pollutant emitted by mining activities is Pb. Regarding the percentages of Cd transfer from soil to grass and from grass to milk, they were higher than those observed for Pb; similarly, the bioaccumulation percentage of Cd (from soil to milk) was 41 times more (3.30/0.08) than that of Pb, a result that would indicate that Cd would be the main toxic metal in the milk produced in the study areas. As demonstrated in our data, the bioaccumulation of these metals depends on their concentration in soil and pasture [97], which is affected by other factors, including the level of soil fertility, content of organic matter, and biological peculiarities of the plants [98,99,100].

### 4.2. Weekly Intake (WI) of Pb and CD in a Peruvian Population Aged 2–85 Years Due to Consumption of Milk Produced in the Mantaro River Headwaters

Evaluating the content of heavy metals in foods and determining dietary exposure and potential health risks allows for the generation of scientific evidence for decision-making. The EDI and WI values of Pb and Cd were calculated for people aged 2–85 years old in three consumption scenarios. The EDI values for Pb for men and women were 0.294 to 7.00 and 0.354 to 7.356; for Cd, the values were 0.241 to 5.742 and 0.290 to 6.034 μg/kg/day, respectively (Table 2). The EDI values for these metals in children and adolescents were higher than the RfD values of Pb and Cd [72,74,101,102].

In the minimum, average, and maximum milk intake scenarios, in people under 9, 12, and 13 years of age, the Pb WI exceeded the tolerable weekly intake (TWI) below the TWI in older people (Figure 2). In the case of Cd in the three milk intake scenarios, the WI greatly exceeded the tolerable weekly intake (TWI) values in people aged 2–19 years and was also higher than the TWI in people older than 60 years with maximum milk intake (Figure 2). As evidenced, infants are a high-risk group for exposure to toxic metals [31].

### 4.3. Target Risk Coefficient (THQ) and Risk Index (HI) in a Peruvian Population Aged 2–85 Years Due to Consumption of Milk Produced in the Mantaro River Headwaters

Figure 3 and Figure 4 show that the THQs for Pb and Cd were >1 in children and young people because they have a lower weight and higher milk intake than adults, a result indicative of a relationship between THQ values and age [29]. The THQs for Pb and Cd in our study are higher than those reported in other regions of the world, such as in Alexandria, Egypt, where the THQs are <1 [103], and Tehran, Iran [104]. In Guelma, Algeria, the THQ values for Pb and Cd were >1 for infants [82]. A recent global study reports that, in ten of seventy regions, the THQ for Pb was >1, and in six of fifty-nine, the THQ for Cd was also >1, a result associated with the presence of several fatal diseases [7].

Regarding the HI, Figure 5 shows that the greater the milk intake, the higher the risk index. Additionally, in addition to people under 20 years of age, the HI value in older adults exceeds the value of 1. The contributions of the THQ of Pb and Cd to the HI were 74.4% and 25.6%, respectively. The HI values for people aged 2 to 85 in the minimum consumption scenario were 0.33 to 7.74. For average milk consumption, the values were between 0.51 and 9.68, and in the maximum intake scenario, the values were between 0.75 and 11.61, with the highest values recorded at a younger age, a result that is related to other studies [29].

The HI values for the three levels of milk intake were assessed as being >1 for those younger than 19 years of age and those over 60 years of age, and these results indicate that the milk produced in the central Andes of Peru is unsuitable for human consumption. This is more consequential for small children who consume more milk in relation to their body weight than adults [29]. As such, the production of milk is safe for human consumption, if adequate control is guaranteed and trace metal remediation strategies are applied in the region [105]. Trace metals have accumulated for extended periods in soils in the valley from both the mining–metallurgy industries and the agricultural application of fertilizer [9,18,85]. This has also been reported in related studies elsewhere [40,85]. At the same time, control of the use of phosphorus agrochemicals must be enforced in agriculture [40], since these agrochemicals are the principal source of Cd [88,106].

In Guelma, an HI > 1 has been reported, and the contributions of each metal to the HI due to milk consumption generally followed a descending order for Pb, Cr, Cd, Ni, Zn, Cu, and Fe with values of 68.19%, 15.39%, 6.91%, 4.94%, 3.42%, 0.88%, and 0.28%, respectively, registering a potential risk of heavy metals, especially Pb, for infants [82]. In a recent study conducted in industrial areas of China, the HI values for people aged 3 to 69 years were in the range of 0.0145 to 0.0967, much less than the threshold of 1, and consumption of this milk will not cause adverse effects over time throughout life [29].

Our results indicate that milk produced in the study area would not be suitable for consumption for people < 20 years of age, who consume milk in high quantities. This is evidence that is valuable for monitoring, reducing, and or preventing the adverse health effects of milk intake [7]. Finally, it must be considered that milk is not the only food product involved. Milk and derivative dairy products represent a small proportion of the daily diet, and the non-carcinogenic risk identified in this study from trace metals contamination could be compounded by accumulation in other foodstuffs, greatly increasing the overall risk to health in the wider population and perhaps especially in infants and adolescents.

### 4.4. Objective Risk Coefficient (THQ) and Risk Index (HI) in a Peruvian Population Aged 2–85 Years Due to Consumption of Milk Produced in the Central Plateau at Various Times of the Year

When comparing the THQ and HI values in the scenarios of minimum, average, and maximum milk intake at differing times in the year, we see a slight increase in Pb and Cd contamination in milk during the dry season (Figure 5, Figure 6 and Figure 7). 

The Hazard Index (HI), the health risk associated with higher levels of Pb and Cd in milk produced in the Mantaro Valley, is slightly higher for both trace metals in the dry season. The HI value is higher than 1 for all ages in the scenario of maximum milk consumption, while in the rainy season, in the scenario of maximum milk consumption, the HI is higher than 1 for people under 23 years old and those over 60 years old. However, the HI for the rest of the population (24–59 years) is close to 1, which would suggest a slight increase in pollution in the dry season. These results corroborate observations indicating that, in the dry season, elevated trace metal contamination is recorded. This is the case along the Nzhelele River in South Africa [107], where trace metals accumulate from irrigation waters in soils and crop plants [108]. In the dry season, the trace metal concentration is elevated because of increased evaporation from water bodies [109]. Consequently, this is most likely the principal cause of seasonally elevated levels of trace metal contamination in dairy products in the region and clearly requires further investigation. This extensive study provides a unique insight into the dispersion and concentration of trace metals in agroecological systems, derived from mining activity and the use and misuse of agricultural inputs. It further underlines the necessity of further investigation to develop strategies and regulatory frameworks to ensure that environmental contamination is minimized and the adverse impacts on human health are avoided.

### 4.5. Implication of Pb and Cd Intake on Human Health

Scientific information indicates that elevated Pb and Cd concentrations impair neurological development and function in human populations. Simultaneous ingestion has several effects on the CNS, and in addition to causing cognitive impairment, it can impact social behavior and be responsible for increased depressive disorders [110,111,112,113], generalized anxiety disorders, panic/agoraphobia, and psychological conditions that could even lead to suicide [114,115,116,117]. Clinical research reports that patients with depressive disorders and panic attacks have high concentrations of Pb and Cd in their system [118]. Although Pb and Cd are considered class 1 toxicants, few studies evaluate the psychological risks due to their presence in the body [119]. The increase in the level of Pb in the body increases the rate of hyperactivity and anxiety [120], and the ingestion of Cd in children can cause short- and long-term cognitive deficits [121], making it necessary to control the levels of heavy metals and metalloids consumed in foods.

In Peru, in an evaluation of mental health and its relationship with exposure to Pb, Cd, and As in children and adults near the Las Bambas mining project in Apurímac, children under three years of age reported detrimental effects on psychomotor development [122]. The Stanford–Binet test in children from 3 to 12 years old reported cases of mild mental disability (2.1%) and borderline mental disability (3.1%) [123]. In those over 12 years of age, 34% reported anxiety and 18% reported depression [123]. In the constitutional province of Callao, in an evaluation of the intellectual and anxiety levels in children chronically intoxicated with Pb, which categorized them as Not Intoxicated (<10 mg/dL) and Intoxicated (>10 mg/dL), a lower IQ and a higher level of anxiety were reported in Intoxicated children [122].

It is also known that simultaneous exposure to Pb, Cd, and As during pregnancy and breastfeeding can affect neurodevelopment in the first years of life and can lead to autism spectrum disorders, attention deficit hyperactivity disorders, anxiety, and depression [112,113]. However, there are not enough studies on the risks of these contaminants for mental health, so it is necessary to monitor the levels of these toxins in foods and implement public health policies to protect fetuses and young children from exposure to these contaminants.

The results of this study demonstrate the potential risk of consuming milk contaminated with Pb and Cd in a Peruvian population, with the infant population being the most at risk. However, more research into the levels of trace metal contamination in different dairy regions of the country is essential, and it is important to study these problems clinically and epidemiologically, identifying the possible causes of milk contamination and taking corrective measures.

## 5. Conclusions

Milk produced in the three zones of the Mantaro River headwaters in the central Andes of Peru has Pb and Cd contents significantly above recommended maximum limits, giving risk indices higher than 1 in the population under 20 years of age in the minimum and average intake scenarios. In the maximum intake scenario, a potential risk was also identified in those over 60 years, indicating a risk of adverse health consequences. The main routes of entry of Pb into the food chain are atmospheric aerosols in dry air or clouds from the mining–metallurgy industries in the La Oroya complex, and for Cd, these are contaminated irrigation waters and phosphate rock fertilizers used in agriculture. These concentrate in soils, pasture grasslands, and milk as a typical process of bioaccumulation. The findings of this study indicate the need for careful monitoring and control of the production of dairy products throughout the Andes. Additionally, data indicate the need for both the development and application of guidelines for monitoring water quality, soil, and milk for the transfer and bioaccumulation of trace metals to guarantee milk production that is safe for human consumption. In general, no marked differences were observed in the level of contamination by Pb and Cd in milk between the dry and rainy seasons; however, more studies should be carried out to better understand the dynamics of heavy metal contamination in the soil–pasture–milk system and the risk to the health of consumers of milk produced in the central Andes of Peru.

## Figures and Tables

**Table 1:** The concentration of Pb and Cd in soil, pasture, and milk (mg/kg) and percentage of transfer and bioaccumulation.

		Pb			Cd		
		Soil	Pasture	Milk	Soil	Pasture	Milk
Paccha	Rain	217.81 ± 39.48	20.10 ± 3.92	0.581 ± 0.018	4.848 ± 0.791	0.601 ± 0.084	0.020 ± 0.007
	Dry	217.71 ± 37.71	20.10 ± 3.04	0.573 ± 0.021	4.605 ± 0.783	0.564 ± 0.074	0.017 ± 0.007
	Average	217.76 ^a^	20.10 ^b^	0.577 ^c^	4.726 ^a^	0.582 ^b^	0.018 ^c^
Mantaro	Rain	56.11 ± 14.31	4.80 ± 1.91	0.015 ± 0.003	10.05 ± 4.05	2.846 ± 0.93	0.603 ± 0.050
	Dry	55.00 ± 3.96	3.78 ± 1.20	0.014 ± 0.003	5.74 ± 1.17	2.853 ± 1.37	0.407 ± 0.092
	Average	55.55 ^a^	4.29 ^b^	0.015 ^c^	7.896 ^a^	2.849 ^b^	0.505 ^c^
Yauris	Rain	652.35 ± 143	23.17 ± 7.49	0.064 ± 0.008	7.089 ± 2.16	0.248 ± 0.35	0.015 ± 0.005
	Dry	354.71 ± 137	10.33 ± 6.02	0.055 ± 0.028	0.008 ± 0.006	0.002 ± 0.0007	0.006 ± 0.004
	Average	503.53 ^a^	16.75 ^b^	0.060 ^c^	3.549 ^a^	0.125 ^b^	0.011 ^c^
Mean	Rain	308.76 ± 267	16.02 ± 9.48	0.220 ± 0.25	7.329 ± 3.41	1.231 ± 1.29	0.213 ± 0.28
	Dry	209.14 ± 147	11.40 ± 7.80	0.214 ± 0.257	3.451 ± 2.62	1.139 ± 1.46	0.143 ± 0.20
	Average	258.95 ^a^	13.71 ^b^	0.217 ^c^	5.390 ^a^	1.185 ^b^	0.178 ^c^
MPL, mg/kg		70	30	0.02	1.4	1.0	0.0026
Transfer percentage		100.00	5.30	1.58	100.00	21.99	15.00
Bioaccumulation percentage		100.00		0.08	100.00		3.30

^a,b,c^ Average values per site per element with different letters vary statistically (*p* < 0.01). MPL: Maximum Permissible Limit. Transfer percentage: from soil to grass and from grass to milk. Bioaccumulation percentage: soil to milk.

**Table 2:** EDI values of Pb and Cd for average milk consumption in people aged 2–85 years.

	EDI Lead		EDI Cadmium		WI Lead		WI Cadmium		TWI	
Age	Man	Woman	Man	Woman	Man	Woman	Man	Woman	Lead	Cadmium
2	7.000	7.356	5.742	6.034	607.60	607.60	498.40	498.40	310.00	71.92
5	4.849	4.989	3.978	4.092	607.60	607.60	498.40	498.40	447.50	103.82
10	3.519	3.495	2.886	2.867	729.12	729.12	598.08	598.08	740.00	171.68
15	2.099	2.201	1.721	1.805	759.50	759.50	623.00	623.00	1292.50	299.86
20	0.345	0.430	0.283	0.353	145.82	161.01	119.62	132.08	1507.50	349.74
25	0.318	0.408	0.260	0.335	145.82	161.01	119.62	132.08	1640.00	380.48
30	0.314	0.386	0.258	0.317	145.82	161.01	119.62	132.08	1657.50	384.54
35	0.313	0.388	0.257	0.318	145.82	161.01	119.62	132.08	1662.50	385.70
40	0.294	0.363	0.241	0.298	142.79	156.46	117.12	128.34	1732.50	401.94
45	0.306	0.365	0.251	0.300	142.79	156.46	117.12	128.34	1665.00	386.28
50	0.302	0.368	0.248	0.302	142.79	156.46	117.12	128.34	1690.00	392.08
55	0.301	0.369	0.247	0.303	142.79	156.46	117.12	128.34	1695.00	393.24
60	0.402	0.484	0.330	0.397	182.28	200.51	149.52	164.47	1620.00	375.84
65	0.404	0.508	0.332	0.417	182.28	200.51	149.52	164.47	1610.00	373.52
70	0.439	0.526	0.360	0.431	182.28	200.51	149.52	164.47	1482.50	343.94
75	0.418	0.553	0.343	0.454	182.28	200.51	149.52	164.47	1557.50	361.34
80	0.463	0.541	0.379	0.444	182.28	200.51	149.52	164.47	1407.50	326.54
85	0.468	0.579	0.384	0.475	182.28	200.51	149.52	164.47	1390.00	322.48

EDI: Estimated daily intake (µg/kg bw/day). WI: Weekly intake (µg/week). TWI: Tolerable weekly intake.

**Table 3:** THQ values of Pb and Cd and HI for average milk consumption in people aged 2–85.

	THQ Lead		THQ Cadmium		HI	
Age	Man	Woman	Man	Woman	Man	Woman
2	2.00	2.10	5.74	6.03	7.74	8.14
5	1.39	1.43	3.98	4.09	5.36	5.52
10	1.01	1.00	2.89	2.87	3.89	3.87
15	0.60	0.63	1.72	1.81	2.32	2.43
20	0.10	0.12	0.28	0.35	0.38	0.48
25	0.09	0.12	0.26	0.33	0.35	0.45
30	0.09	0.11	0.26	0.32	0.35	0.43
35	0.09	0.11	0.26	0.32	0.35	0.43
40	0.08	0.10	0.24	0.30	0.33	0.40
45	0.09	0.10	0.25	0.30	0.34	0.40
50	0.09	0.11	0.25	0.30	0.33	0.41
55	0.09	0.11	0.25	0.30	0.33	0.41
60	0.11	0.14	0.33	0.40	0.44	0.54
65	0.12	0.15	0.33	0.42	0.45	0.56
70	0.13	0.15	0.36	0.43	0.49	0.58
75	0.12	0.16	0.34	0.45	0.46	0.61
80	0.13	0.15	0.38	0.44	0.51	0.60
85	0.13	0.17	0.38	0.47	0.52	0.64

## Data Availability

Data supporting the reported results can be requested from the corresponding author.

## References

[1] (2015). Milk Fact Nutritional Facts. Nutritional Components of Milk. http://milkfacts.info/Nutrition%20Facts/Nutritional%20Components.htm.

[2] Farré R. (2015). La leche y los productos lácteos: Fuentes dietéticas de calcio. Nutr. Hosp..

[3] González-Rodríguez R., Jiménez-Escobar I., Gutiérrez-Castrellón P. (2020). Microbiota de la leche humana y su impacto en la salud humana. Gac. Med. Mex..

[4] Givens D.I. (2020). MILK Symposium review: The importance of milk and dairy foods in the diets of infants, adolescents, pregnant women, adults, and the elderly. J. Dairy Sci..

[5] Fukase E., Martin W. (2020). Economic growth, convergence, and world food demand and supply. World Dev..

[6] Chandravanshi L., Shiv K., Kumar S. (2021). Developmental toxicity of cadmium in infants and children: A review. Environ. Anal. Health Toxicol..

[7] Boudebbouz A., Boudalia S., Bousbia A., Habila S., Boussadia M.I., Gueroui Y. (2021). Heavy metals levels in raw cow milk and health risk assessment across the globe: A systematic review. Sci. Total Environ..

[8] Kwon J.C., Nejad Z.D., Jung M.C. (2017). Arsenic and heavy metals in paddy soil and polished rice contaminated by mining activities in Korea. Catena.

[9] Castro-Bedriñana J., Chirinos-Peinado D., García-Olarte E., Quispe-Ramos R., Gordillo-Espinal S. (2021). Lead transfer in the soil-root-plant system in a highly contaminated Andean area. PeerJ.

[10] Zhou X., Zheng N., Su C., Wang J., Soyeurt H. (2019). Relationships between Pb, As, Cr, and Cd in individual cows’ milk and milk composition and heavy metal contents in water, silage, and soil. Environ. Pollut..

[11] Kuppusamy S., Palanisami T., Megharaj M., Venkateswarlu K., Naidu R., de Voogt P. (2016). In-situ remediation approaches for the management of contaminated sites: A comprehensive overview. Reviews of Environmental Contamination and Toxicology.

[12] Nkwunonwo U.C., Odika P.O., Onyia N.I. (2020). A Review of the Health Implications of Heavy Metals in Food Chain in Nigeria. Sci. World J..

[13] Wei J., Cen K. (2020). Contamination and health risk assessment of heavy metals in cereals, legumes, and their products: A case study based on the dietary structure of the residents of Beijing, China. J. Clean. Prod..

[14] Atkinson N.J., Urwin P.E. (2012). The interaction of plant biotic and abiotic stresses: From genes to field. J. Exp. Bot..

[15] González-Montaña J.R., Senís E., Gutiérrez A., Prieto F. (2012). Cadmium and lead in bovine milk in the mining area of the Caudal River (Spain). Environ. Monit. Assess..

[16] Shamimeh A.S., Narges S.O., Zeinab D., Zahra H., Arvin A., Ehsan S., Moein B. (2023). A comprehensive image of environmental toxic heavy metals in red meat: A global systematic review and meta-analysis and risk assessment study. Sci. Total Environ..

[17] Castro J., López de Romaña D., Bedregal P., López de Romaña G., Chirinos D. (2013). Lead and cadmium in maternal blood and placenta in pregnant women from a mining-smelting zone of Peru and transfer of these metals to their newborns. J. Toxicol. Environ. Health Sci..

[18] Castro-Bedriñana J., Chirinos-Peinado D., Ríos-Ríos E., Machuca-Campuzano M., Gómez-Ventura E. (2021). Dietary risk of milk contaminated with lead and cadmium in areas near mining-metallurgical industries in the Central Andes of Peru. Ecotoxicol. Environ. Saf..

[19] Karimi A., Naghizadeh A., Biglari H., Peirovi R., Ghasemi A., Zarei A. (2020). Assessment of human health risks and pollution index for heavy metals in farmlands irrigated by effluents of stabilization ponds. Environ. Sci. Pollut. Res..

[20] Acevedo S.C., Loaiza D.C., Mesa G.P. (2013). Determinación de cadmio en leches crudas usando un biosensor amperométrico. Rev. Lasallista Investig..

[21] Assi M.A., Hezmee M.N., Haron A.W., Sabri M.Y., Rajion M.A. (2016). The detrimental effects of lead on human and animal health. Vet. World.

[22] Sharifi-Rad J., Mnayer D., Roointan A., Shahri F., Ayatollahi S.A., Sharifi-Rad M., Molaee N., Sharifi-Rad M. (2016). Antibacterial activities of essential oils from Iranian medicinal plants on extended-spectrum β-lactamase-producing Escherichia coli. Cell. Mol. Biol..

[23] López-Rodríguez G., Galván M., González-Unzaga M., Hernández-Ávila J., Pérez-Labra M. (2017). Blood toxic metals and hemoglobin levels in Mexican children. Environ. Monit. Assess..

[24] Minkina T.M., Mandzhieva S.S., Burachevskaya M.V., Bauer T.V., Sushkova S.N. (2018). Method of determining loosely bound compounds of heavy metals in the soil. Methods X.

[25] Castro-González N.P., Calderón-Sánchez F., Pérez-Sato M., Soní-Guillermo E., Reyes-Cervantes E. (2019). Health risk due to chronic heavy metal consumption via cow’s milk produced in Puebla, Mexico, in irrigated wastewater areas. Food Addit. Contam. Part B.

[26] Ibrahim M.I., Mohamed L.A., Mahmoud M.G., Shaban K.S., Fahmy M.A., Ebeid M.H. (2019). Potential ecological hazards assessment and prediction of sediment heavy metals pollution along the Gulf of Suez, Egypt. Egypt J. Aquat. Res..

[27] Zhou Q., Fellows A., Flerchinger G.N., Flores A.N. (2019). Examining Interactions Between and Among Predictors of Net Ecosystem Exchange: A Machine Learning Approach in a Semi-arid Landscape. Sci. Rep..

[28] Affum A.O., Osae S.D., Kwaansa-Ansah E.E., Miyittah M.K. (2020). Quality assessment and potential health risk of heavy metals in leafy and non-leafy vegetables irrigated with groundwater and municipal-waste-dominated stream in the Western Region, Ghana. Heliyon.

[29] Su C., Gao Y., Qu X., Zhou X., Yang X., Huang S., Han L., Zheng N., Wang J. (2021). The Occurrence, Pathways, and Risk Assessment of Heavy Metals in Raw Milk from Industrial Areas in China. Toxics.

[30] Varol M., Sünbül M.R. (2020). Macroelements and toxic trace elements in muscle and liver of fish species from the largest three reservoirs in Turkey and human risk assessment based on the worst-case scenarios. Environ. Res..

[31] Amarh F.A., Agorku E.S., Voegborlo R.B., Ashong G.W., Atongo G.A. (2023). Health risk assessment of some selected heavy metals in infant food sold in Wa, Ghana. Heliyon.

[32] Hama Aziz K.H., Mustafa F.S., Omer K.M., Hama S., Hamarawf R.F., Rahman K.O. (2023). Heavy metal pollution in the aquatic environment: Efficient and low-cost removal approaches to eliminate their toxicity: A review. RSC Adv..

[33] Donnachie R.L., Johnson A.C., Moeckel C., Pereira M.G., Sumpter J.P. (2014). Using risk-ranking of metals to identify which poses the greatest threat to freshwater organisms in the UK. Environ. Pollut..

[34] Lü Q., Xiao Q., Wang Y., Wen H., Han B., Zheng X., Lin R. (2020). Risk assessment and hotspots identification of heavy metals in rice: A case study in Longyan of Fujian province, China. Chemosphere.

[35] Chirinos-Peinado D., Castro-Bedriñana J., Ríos-Ríos E., Mamani-Gamarra G., Quijada-Caro E., Huacho-Jurado A., Nuñez-Rojas W. (2022). Lead and Cadmium Bioaccumulation in Fresh Cow’s Milk in an Intermediate Area of the Central Andes of Peru and Risk to Human Health. Toxics.

[36] Castro-Bedriñana J., Chirinos-Peinado D., Ríos-Ríos E., Castro-Chirinos G., Chagua-Rodríguez P., De La Cruz-Calderón G. (2023). Lead, Cadmium, and Arsenic in Raw Cow’s Milk in a Central Andean Area and Risks for the Peruvian Populations. Toxics.

[37] NTP (2013). Norma Técnica Peruana N° 202.112, 1998 (Revisada el 2013). Leche y Productos Lácteos. Leche Cruda. Muestreo de Productos Lácteos, Instrucción General. 1ra Edición. https://www.inacal.gob.pe/repositorioaps/data/1/1/6/jer/pntp-en-dp/files/02-05%20de%20febrero.pdf.

[38] García-Gallegos E., Hernández-Acosta E., García-Nieto E., Acevedo-Sandoval O. (2011). Contenido y traslocación de plomo en avena (*Avena sativa*, L.) y habaA (*Vicia faba*, L.) de un suelo contaminado. Rev. Chapingo Ser. Cienc. For. Ambient.

[39] MINAM (2014). Guía Para Muestreo de Suelo. Ministerio del Ambiente-Perú. https://www.minam.gob.pe/wp-content/uploads/2014/04/GUIA-MUESTREO-SUELO_MINAM1.pdf.

[40] Martín A.P., Turnbull R.E., Rissmann C.W., Rieger P. (2017). Heavy metal and metalloid concentrations in soils under pasture of Southern New Zealand. Geoderma Reg..

[41] Bidar G., Pruvot C., Garçon G., Verdin A., Shirali P., Douay F. (2009). Seasonal and annual variations of metal uptake, bioaccumulation, and toxicity in Trifolium repens and Lolium perenne growing in a heavy metal-contaminated field. Environ. Sci. Pollut. Res..

[42] Chirinos-Peinado D., Castro-Bedriñana J., Ríos-Ríos E., Castro-Chirinos G., Quispe-Poma Y. (2023). Lead, Cadmium, and Arsenic in Raw Milk Produced in the Vicinity of a Mini Mineral Concentrator in the Central Andes and Health Risk. Biol. Trace Elem. Res..

[43] Latimer G.W. (2016). AOAC Official Method 973.35 Lead in Evaporated Milk Atomic Absorption Spectrophotometric Method.

[44] Anjos D.C., Hernandez F.F., Bañuelos G.S., Dangi S.R., Tirado-Corbalá R., da Silva F.N., Filho P.F. (2018). Microbial community and heavy metals content in soils along the Curu River in Ceará, Brazil. Geoderma Reg..

[45] MINAM (2017). Resolución Ministerial No. 182-2017-MINAM. Estándares Nacionales de Calidad Ambiental (ECA), Para Suelo. https://goo.su/5jv9Vrk.

[46] Boularbah A., Schwartz C., Bitton G., Morel J.L. (2006). Heavy metal contamination from mining sites in South Morocco: 1. Use of a biotest to assess metal toxicity of tailings and soils. Chemosphere.

[47] Boularbah A., Schwart C., Bitton G., Aboudrar W., Ouhammou A., Morel J.L. (2006). Heavy metal contamination from mining sites in South Morocco: 2. Assessment of metal accumulation and toxicity in plants. Chemosphere.

[48] Kabata-Pendias A., Mukherjee A.B. (2007). Trace Elements from Soil to Human.

[49] OJEU (2013). Commission Regulation (EU) No 1275/2013. Official Journal of the European Union. https://acortar.link/OssuRK.

[50] (1995). Codex Alimentarius General Standard for Contaminants and Toxins in Food and Feed.

[51] European Commission (2006). Commission Regulation (EC) No 1881/2006 of 19 December 2006 setting maximum levels for certain contaminants in foodstuffs. Off. J. Eur. Union..

[52] IDF (1979). Standard I. International Dairy Federation Bulletin, Chemical Residues in Milk and Milk Products. IDF Document.

[53] Papaioannou D., Kalavrouziotis I.K., Koukoulakis P.H., Papadopoulos F., Psoma P. (2018). Interrelationships of metal transfer factor under wastewater reuse and soil pollution. J. Environ. Manag..

[54] Drozdova I., Alekseeva-Popova N., Dorofeyev V., Bech J., Belyaeva S., Roca N. (2019). A comparative study of the accumulation of trace elements in Brassicaceae plant species with phytoremediation potential. Appl. Geochem..

[55] USEPA (2015). Integrated Risk Information System.

[56] Muñoz O., Zamorano P., Garcia O., Bastias J.M. (2017). Arsenic, cadmium, mercury, sodium, and potassium concentrations in common foods and estimated daily intake of the population in Valdivia (Chile) using a total diet study. Food Chem. Toxicol..

[57] Zheng S., Wang Q., Yuan Y., Sun W. (2020). Human health risk assessment of heavy metals in soil and food crops in the Pearl River Delta urban agglomeration of China. Food Chem..

[58] Dror D.K., Allen L.H. (2013). Dairy product intake in children and adolescents in developed countries: Trends, nutritional contribution, and a review of association with health outcomes. Nutr. Rev..

[59] Singh G.M., Micha R., Khatibzadeh S., Shi P., Lim S., Andrews K.G., Engell R.E., Ezzati M., Mozaffarian D. (2015). Global, regional, and national consumption of sugar-sweetened beverages, fruit juices, and milk: A systematic assessment of beverage intake in 187 countries. PLoS ONE.

[60] Aparco J.P., Bautista-Olórtegui W., Astete-Robilliard L., Pillaca J. (2016). Evaluación del estado nutricional, patrones de consumo alimentario y de actividad física en escolares del Cercado de Lima. Rev. Peru. Med. Exp. Salud Publica.

[61] Grenov B., Larnkjær A., Mølgaard C., Michaelsen K.F. (2020). Role of milk and dairy products in growth of the child. Nestle Nutr. Inst. Workshop.

[62] CENAN-INEI (2011). Estado Nutricional en el Perú. Componente Nutricional ENAHO-CENANz-INS. Ministerio de Salud, Lima, Perú. https://bvs.minsa.gob.pe/local/MiNSA/1843.pdf.

[63] Atique Ullah A.K.M., Maksud M.A., Khan S.R., Lutfa L.N., Quraishi S.B. (2017). Dietary intake of heavy metals from eight highly consumed species of cultured fish and possible human health risk implications in Bangladesh. Toxicol. Rep..

[64] Christophoridis C., Kosma A., Evgenakis E., Bourliva A., Fytianos K. (2019). Determination of heavy metals and health risk assessment of cheese products consumed in Greece. J. Food Compos. Anal..

[65] Bandara S.B., Towle K.M., Monnot A.D. (2020). A human health risk assessment of heavy metal ingestion among consumers of protein powder supplements. Toxicol. Rep..

[66] EFSA (2012). Lead dietary exposure in the European population. EFSA J..

[67] EFSA (2012). Cadmium dietary exposure in the European population. EFSA J..

[68] JECFA (2011). Joint FAO/WHO Expert Committee on Food Additives (2011) Evaluation of Certain Food Additives and Contaminants. 73 Report, 2010.

[69] JWH, Joint and World Health Organization (2012). Safety Evaluation of Certain Food Additives and Contaminants: Prepared by the Seventy Fourth Meeting of the Joint FAO/WHO Expert Committee on Food Additives (JECFA).

[70] EFSA (2011). EFSA Panel on Contaminants in the Food Chain (CONTAM); Scientific opinion on tolerable weekly intake for cadmium. EFSA J..

[71] Rahmani J., Fakhri Y., Shahsavani A., Bahmani Z., Urbina M.A., Chirumbolo S., Keramati H., Moradi B., Bay A., Bjørklund G. (2018). A systematic review and meta-analysis of metal concentrations in canned tuna fish in Iran and human health risk assessment. Food Chem. Toxicol..

[72] USEPA (2011). USEPA Regional Screening Level (RSL) Summary Table.

[73] USEPA (2012). United States Environmental Protection Agency. Reference Dose (RfD): Description and Use in Health Risk Assessments. https://www.epa.gov.

[74] Khan N., Jeong I.S., Hwang I.M., Kim J.S., Choi S.H., Nho E.Y., Choi J.Y., Park K.S., Kim K.S. (2014). Analysis of minor and trace elements in milk and yogurts by inductively coupled plasma-mass spectrometry (ICP-MS). Food Chem..

[75] Zhuang P., McBride M.B., Xia H., Li N., Li Z. (2009). Health risk from heavy metals via consumption of food crops in the vicinity of Dabaoshan mine, South China. Sci. Total Environ..

[76] Liu X., Song Q., Tang Y., Li W., Xu J., Wu J., Wang F., Brookes P.C. (2013). Human health risk assessment of heavy metals in soil-vegetable system: A multi-medium analysis. Sci. Total Environ..

[77] Singh D., Kumar A. (2020). Quantification of metal uptake in Spinacia oleracea irrigated with water containing a mixture of CuO and ZnO nanoparticles. Chemosphere.

[78] ATSDR (2012). Public Health Statement for Cadmium. Agency for Toxic Substances and Disease Registry (ATSDR). http://www.atsdr.cdc.gov/toxprofiles/tp.asp?id=48&tid=15.

[79] Castro J., Chirinos-Peinado D., Peñaloza R. (2020). Lead bioaccumulation in root and aerial part of natural and cultivated pastures in highly contaminated soils in Central Andes of Peru. Adv. Sci. Technol. Eng. Syst. J..

[80] Hou Q., Yang Z., Ji J., Yu T., Chen G., Li J., Xia X., Zhang M., Yuan X. (2014). Annual net input fluxes of heavy metals of the agroecosystem in the Yangtze River delta, China. J. Geochem. Explor..

[81] Kozhanova N., Sarsembayeva N., Lozowicka B., Kozhanov Z. (2021). Seasonal content of heavy metals in the “soil-feed-milk-manure” system in horse husbandry in Kazakhstan. Vet. World.

[82] Boudebbouz A., Boudalia S., Bousbia A., Gueroui Y., Boussadia M.I., Chelaghmia M.L., Zebsa R., Affoune A.M., Symeon G.K. (2022). Determination of Heavy Metal Levels and Health Risk Assessment of Raw Cow Milk in Guelma Region, Algeria. Biol. Trace Elem. Res..

[83] Norouzirad R., González-Montaña J.R., Martínez-Pastor F., Hosseini H., Shahrouzian A., Khabazkhoob M., Ali Malayeri F., Moallem Bandani H., Paknejad M., Foroughi-nia B. (2018). Lead and cadmium levels in raw bovine milk and dietary risk assessment in areas near petroleum extraction industries. Sci. Total Environ..

[84] Khan S., Cao Q., Zheng Y.M., Huang Y.Z., Zhu Y.G. (2008). Health risks of heavy metals in contaminated soils and food crops irrigated with wastewater in Beijing, China. Environ. Pollut..

[85] Chirinos-Peinado D., Castro-Bedriñana J., García-Olarte E., Quispe-Ramos R., Gordillo-Espinal E. (2021). Transfer of lead from soil to pasture grass and milk near a metallurgical complex in the Peruvian Andes. Transl. Anim. Sci..

[86] Tepanosyan G., Sahakyan L., Belyaeva O., Maghakyan N., Saghatelyan A. (2017). Human health risk assessment and riskiest heavy metal origin identification in urban soils of Yerevan, Armenia. Chemosphere.

[87] Ha T.T., Tu V., Tam K.B., Ha T.H. (2019). Accumulation of arsenic and heavy metals in native and cultivated plant species in a lead recycling area in Vietnam. Minerals.

[88] European-Union (2015). Commission Regulation (EU) 2015/1005 of 25 June 2015 Amending Regulation (EC) N° 1881/2006 as Regards Maximum Levels of Lead in Certain Foodstufs. Off. J. Eur. Union.

[89] Codex Alimentarius (2011). Guidelines for risk analysis of foodborne antimicrobial resistance. CAC/GL.

[90] Schipper L.A., Sparling G.P., Fisk L.M., Dodd M.B., Power I.L., Littler R.A. (2011). Rates of accumulation of cadmium and uranium in a New Zealand hill farm soil as a result of long-term use of phosphate fertilizer. Agric. Ecosyst. Environ..

[91] Zhang F., Li Y., Yang M., Li E. (2012). Content of heavy metals in animal feeds and manures from farms of different scales in Northeast China. Int. J. Environ. Res. Public Health.

[92] Su C., Liu H., Qu X., Zhou X., Gao Y., Yang H., Zheng N., Wang J. (2020). Heavy metals in raw milk and dietary exposure assessment in the vicinity of leather-processing plants. Biol. Trace Elem. Res..

[93] Eleboudi A.A., El-Makarem H.A., Hadour H.H.A. (2017). Heavy metals residues in some dairy products. Alex. J. Vet. Sci..

[94] Shahbazi Y., Ahmadi F., Fakhari F. (2016). Voltammetric determination of Pb, Cd, Zn, Cu, and Se in milk and dairy products collected from Iran: An emphasis on permissible limits and risk assessment of exposure to heavy metals. Food Chem..

[95] Muhib I., Alamgir M., Chowdhury Z., Easha N.J., Rahman M. (2016). Investigation of heavy metal contents in Cow milk samples from area of Dhaka, Bangladesh. Int. J. Food. Contam..

[96] Bilandžić N., Sedak M., Čalopek B., Luburić D.D.B., Solomun Kolanović B., Varenina I., Dokić M., Kmetič I., Murati T. (2016). Lead. Concentrations in Raw Cow and Goat Milk. Collected in Rural. Areas of Croatia from 2010 to 2014. Bull. Environ. Contam. Toxicol..

[97] Agrawal S.B., Singh A., Sharma R.K., Agrawal M. (2007). Bioaccumulation of heavy metals in vegetables: A threat to human health Terr. Aquat. Environ. Toxicol..

[98] Chang J.H., Dong C.D., Shen S.Y. (2019). The lead contaminated land treated by the circulation-enhanced electrokinetics and phytoremediation in field scale. J. Hazard. Mater..

[99] Sun K., Wen D., Yang N., Wang K., Li X., Yu L. (2019). Heavy metal and soil nutrient accumulation and ecological risk assessment of vegetable fields in representative facilities in Shandong Province, China. Environ. Monit. Assess..

[100] Sihlahla M., Mouri H., Nomngongo P.N. (2019). Uptake of trace elements by vegetable plants grown on agricultural soils. J. Afr. Earth Sci..

[101] USEPA (2012). EPA Region III Risk-Based Concentration (RBC) Table 2008 Region III, 1650 Arch Street, Philadelphia, Pennsylvania 19103.

[102] Khan M.U., Malik R.N., Muhammad S., Ullah F., Qadir A. (2014). Health Risk Assessment of Consumption of Heavy Metals in Market Food Crops from Sialkot and Gujranwala Districts, Pakistan. Hum. Ecol. Risk Assess. Int. J..

[103] Amer A.A., El-Makarem H.S., El-Maghraby M.A., Abou-Alella S.A. (2011). Lead, cadmium, and aluminum in raw bovine milk: Residue level, estimated intake, and fate during artisanal dairy manufacture. J. Adv. Vet. Anim. Res..

[104] Sharif S., Sohrabvandi S., Mofd V., Javanmardi F., Khanniri E., Mortazavian A.M. (2022). The assessment of lead concentration in raw milk collected from some major dairy farms in Iran and evaluation of associated health risk. J. Environ. Health Sci. Eng..

[105] Tóth G., Hermann T., Da Silva M.R., Montanarella L. (2016). Heavy metals in agricultural soils of the European Union with implications for food safety. Environ. Int..

[106] Bonomelli C., Bonilla C., Valenzuela A. (2003). Efecto de la fertilización fosforada sobre el contenido de cadmio en cuatro suelos de Chile. Pesqui. Agropecu. Bras..

[107] Ahmed A.S., Rahman M., Sultana S., Babu S.M., Sarker M.S. (2019). Bioaccumulation and heavy metal concentration in tissues of some commercial fishes from the Meghna River Estuary in Bangladesh and human health implications. Mar. Pollut. Bull..

[108] Edokpayi J.N., Odiyo J.O., Popoola E.O., Msagati T.A. (2017). Evaluation of temporary seasonal variation of heavy metals and their potential ecological risk in Nzhelele River, South Africa. Open Chem..

[109] Edokpayi J.N., Odiyo J.O., Popoola O.E., Msagati T.A. (2016). Assessment of Trace Metals Contamination of Surface Water and Sediment: A Case Study of Mvudi River, South Africa. Sustainability.

[110] Song S., Liu N., Wang G., Wang Y., Zhang X., Zhao X., Chang H., Yu Z., Liu X. (2023). Sex Specificity in the Mixed Effects of Blood Heavy Metals and Cognitive Function on Elderly: Evidence from NHANES. Nutrients.

[111] Ramírez O.D., González E.D.F., Blanco A.T., Pineda B., Gómez M.S., Marcial Q.J., Carrillo M.P., Pérez de la Cruz V. (2021). Cognitive Impairment Induced by Lead Exposure during Lifespan: Mechanisms of Lead Neurotoxicity. Toxics.

[112] Zaw Y.H., Taneepanichskul N. (2019). Blood heavy metals and brain-derived neurotrophic factor in the first trimester of pregnancy among migrant workers. PLoS ONE.

[113] Skogheim T.S., Weyde K.V., Engel S.M., Aase H., Surén P., Øie G.M., Biele G., Reichborn-Kjennerud T., Caspersen I.H., Hornig M. (2021). Metal and essential element concentrations during pregnancy and associations with autism spectrum disorder and attention-deficit/hyperactivity disorder in children. Environ. Int..

[114] Jaga K., Dharmani C. (2007). The Interrelation between Organophosphate Toxicity and the Epidemiology of Depression and Suicide. Rev. Environ. Health.

[115] Ayuso-Álvarez A., Simón L., Nuñez O., Rodríguez-Blázquez C., Martín-Méndez I., Bel-Lán A., López-Abente G., Merlo J., Fernandez-Navarro P., Galán I. (2019). Association between Heavy Metals and Metalloids in Topsoil and Mental Health in the Adult Population of Spain. Environ. Res..

[116] Berk M., Williams L.J., Andreazza A.C., Pasco J.A., Dodd S., Jacka F.N., Moylan S., Reiner E.J., Magalhaes P.V. (2014). Pop, heavy metal and the blues: Secondary analysis of persistent organic pollutants (POP), heavy metals and depressive symptoms in the NHANES National Epidemiological Survey. BMJ Open.

[117] Theorell T., Hammarström A., Aronsson G., Träskman Bendz L., Grape T., Hogstedt C., Marteinsdottir I., Skoog I., Hall C. (2015). A Systematic Review Including Meta-Analysis of Work Environment and Depressive Symptoms. BMC Public Health.

[118] Jurczak A., Brodowska A., Szkup M., Prokopowicz A., Karakiewicz B., Łój B., Kotwas A., Brodowska A., Grochans E. (2018). Influence of Pb and Cd Levels in Whole Blood of Postmenopausal Women on the Incidence of Anxiety and Depressive Symptoms. Ann. Agric. Environ. Med..

[119] USP (2017). Official from 1 December 2017. The United States Pharmacopeia Convention. https://www.usp.org/sites/default/files/usp/document/our-work/chemical-medicines/key-issues/232-40-35-1s.pdf.

[120] He B., Wang Y., Li S., Zhao Y., Ma X., Wang W., Li X., Zhang Y. (2021). A cross–sectional survey of preschool children: Exploring heavy metal exposure, neurotransmitters, and neurobehavioural relationships and mediation effects. Ecotoxicol. Environ. Saf..

[121] Mann D. (2012). Study Links Cadmium Exposure to Learning Disabilities in Kids. WebMD, LLC. https://www.webmd.com/children/news/20120127/study-links-cadmium-exposure-learning-disabilities-kids.

[122] Vega J., De Coll J., Lermo J., Escobar J., Díaz M., Castro J. (2005). Niveles Intelectuales y Ansiedad en Niños con Intoxicación Plúmbica Crónica. Colegio “María Reiche” Callao-Perú, 2002. An. Fac. Med..

[123] Astete J., Gastañaga M.C., Fiestas V., Oblitas T., Sabastizagal I., Lucero M., Abadíe J.M., Muñoz M.E., Valverde A., Suarez M. (2010). Comunicable Diseases, Mental Health and Exposure to Environmental Pollutants in Population Living Near Las Bambas Mining Project before Exploitation Phase, Peru 2006. Rev. Peru. Med. Exp. Salud Publica.

